# The PTS EIIB Component Drives Strain-Specific Virulence in *Listeria monocytogenes*: Divergent Regulation of Biofilm Formation and Host Infection in High- and Low-Virulence Strains

**DOI:** 10.3390/microorganisms13102274

**Published:** 2025-09-28

**Authors:** Lu Liu, Caixia Liu, Ruixuan Qian, Yatao Qi, Zhongke Yin, Ruifeng Luo, Dongdong Du, Zengqi Liu, Lichao Kang, Jing Wang

**Affiliations:** 1 College of Animal Science and Technology, Shihezi University, Shihezi 832000, China; liulu0626666@163.com (L.L.); liucaixia0402@163.com (C.L.); 15373481428@163.com (R.Q.); 18094801903@163.com (Y.Q.); yzk1849608975@163.com (Z.Y.); 2 Analysis and Testing Center, Xinjiang Academy of Agriculture and Reclamation Sciences, Shihezi 832000, China; luorf2025@163.com (R.L.); dudongdong1201@163.com (D.D.); liuzengqi111@163.com (Z.L.)

**Keywords:** *Listeria monocytogenes*, LIPI-4, phosphotransferase system (PTS), biofilm formation, virulence divergence, metabolic virulence regulation

## Abstract

*Listeria monocytogenes* (*L. monocytogenes*) is a Gram-positive intracellular pathogen capable of causing severe infections. The *Listeria* pathogenicity island 4 (LIPI-4) encodes a phosphotransferase system (PTS) with its EIIB component playing a critical role in carbohydrate phosphorylation and virulence. However, the precise function of EIIB in virulence regulation across diverse pathogenic strains remains unclear. Here, we generated an EIIB deletion mutant (LM873ΔEIIB) and its complemented strain (CLM873ΔEIIB) from the low-virulence strain LM873, and performed comparative analyses with the high-virulence strain LM928 and its corresponding mutants. Deletion of EIIB differentially modulated biofilm formation: suppressing it in LM928 while enhancing it in LM873, accompanied by corresponding transcriptional changes in biofilm-associated and virulence genes. Both mutants exhibited impaired hemolytic activity, whereas motility attenuation was specific to LM928ΔEIIB. At the cellular level, LM873ΔEIIB enhanced adhesion to and invasion of Caco-2 but impaired intracellular proliferation in JEG-3; In contrast, LM928ΔEIIB promoted Caco-2 invasion while attenuating JEG-3 adhesion, invasion, and intracellular replication, as well as reducing invasion and proliferation in RAW264.7 macrophage. Animal experiments demonstrated that EIIB deletion attenuated LM928 colonization in the liver and spleen, but had no significant impact on LM873. Collectively, our findings establish EIIB as a strain-dependent virulence regulator in *L. monocytogenes*, particularly modulating biofilm formation and host–pathogen interactions.

## 1. Introduction

*Listeria monocytogenes* (*L. monocytogenes*) is a Gram-positive intracellular pathogen that causes meningitis and septicemia, particularly in neonates, elderly individuals, and immunocompromised populations [1]. In the United States, *L. monocytogenes* is responsible for approximately 1600 infections annually, with a mortality rate of 19%, largely attributable to its ability to invade host cells and evade immune defenses [2]. Most infections are foodborne, involving a complex pathogenesis with multiple transmission routes [3]. A major public health concern is the pathogen’s capacity to adhere to and form biofilms on food processing surfaces [4], facilitating persistent contamination that resists conventional disinfection and food preservation methods [5,6]. These traits pose significant public health risks and challenge existing food safety controls.

*L. monocytogenes* produces various virulence factors that facilitate epithelial cell adhesion, cell-to-cell spread, intracellular replication, and host barrier penetration [7]. Among these, the LIPI-1 gene cluster is a conserved virulence determinant found in all known serotypes. It contains six key genes: *prf*, *plcA*, *plcB*, *hly*, *mpl*, and *actA*, which are crucial for bacterial invasion, intracellular survival and pathogenicity [8]. LIPI-3 encodes listeriolysin S, a toxin that alters gut microbiota composition and promotes bacterial colonization and invasion [9,10]. In 2016, Maury et al. [11] identified *Listeria* pathogenicity island 4 (LIPI-4) in the hypervirulent CC4 clonal group through multilocus sequence typing (MLST) of clinical and foodborne isolates. LIPI-4 was characterized as a strain-specific virulence factor strongly associated with central nervous system (CNS) and placental infections [11]. A subsequent large-scale analysis of 1027 *L. monocytogenes* isolates from Xinjiang (2014–2017) detected five LIPI-4-positive strains (designated LM461, LM873, LM928, LM2947, and LM5573), all belonging to sequence type 87 (ST87) rather than CC4 [12]. Mouse infection models demonstrated that LIPI-4 deletion in strain LM928 significantly attenuated bacterial colonization, host cell invasion, and intracellular proliferation [13,14], confirming its critical role in virulence. Notably, this key pathogenicity island is also present in *L. innocua*, where the intact LIPI-4 locus is conserved across all isolates despite low sequence similarity (83.7–84.0%) and potentially divergent functions [15]. Comparative virulence assays (median lethal dose, LD_50_) revealed that the low-virulence strain LM873 had an approximately 1000-fold higher LD_50_ than the high-virulence strain LM928, suggesting that LIPI-4-mediated pathogenicity is dependent on virulence background [16]. Similarly, an analysis of 65 *L. monocytogenes* isolates from Ecuador found that while LIPI-4 was predominantly associated with highly virulent strains, partial LIPI-4 gene fragments were also detectable in low-pathogenicity strains (e.g., ST2) [17]. These findings suggest that although LIPI-4 serves as a key marker of hypervirulence, it is not an absolute determinant; its pathogenic effects likely depend on synergistic interactions with other virulence factors or specific genetic contexts. The observed functional divergence of LIPI-4 between high- and low-virulence *L. monocytogenes* strains is not incidental but rather governed by distinct molecular mechanisms.

LIPI-4 is fundamentally comprising the genetic components constituting a phosphoenolpyruvate-dependent phosphotransferase system (PTS), a conserved bacterial mechanism for carbohydrate uptake and phosphorylation [13]. This system includes maltose-6′-P-glucosidase, transcriptional antitermination, uncharacterized protein associated with PTSs, and the membrane-permeable PTS enzymes EIIA, EIIB, and EIIC [11]. These six key components are encoded by the genes *Lm4b_02324*, *Lm4b_02325*, *Lm4b_02326*, *Lm4b_02327*, *Lm4b_02328*, and *Lm4b_02329*, respectively [18]. The EII complex, consisting of the soluble phosphate-transfer domains EIIA and EIIB and the membrane-embedded transporter EIIC, determines carbohydrate specificity and mediates the phosphorylation and internalization of diverse sugars [19]. Beyond its role in sugar metabolism, the PTS acts as a sensor for extracellular stimuli and intracellular metabolic states [20], regulating carbon/nitrogen/phosphate metabolism, chemotaxis, potassium transport, and bacterial virulence [21]. PTS functionality is closely tied to pathogenicity. For example, *Salmonella* with defective PTS components show significantly reduced invasiveness and impaired intracellular survival [22]. Similarly, McAllister et al. [23] reported that deletion of the *cel-PTS* gene in pathogenic *Streptococcus pneumoniae* attenuates murine colonization and virulence across multiple serotypes. Likewise, the mannose PTS (man-PTS) permease in *Listeria monocytogenes* modulates virulence gene expression and pathogenicity [24]. Together, these findings highlight the conserved role of the PTS in virulence regulation among bacterial pathogens.

Studies on *Streptococcus agalactiae* have demonstrated that deletion of the cellobiose (cel) PTS complex significantly impairs cellobiose metabolism and biofilm formation in both high-virulence strain THN0901 and low-virulence strain TFJ0901, while enhancing their adhesion to tilapia brain cells and competitive ability in mixed bacterial environments both in vitro and in vivo [25]. Notably, the cel-EII complex was found to exert significant negative regulation on virulence in the low-virulence strain TFJ0901, an effect absent in the high-virulence strain THN0901 [25]. The EIIB component, which typically forms a membrane-bound fusion with EIIC in PTS, plays a crucial role in carbohydrate phosphorylation [26]. Emerging evidence highlights the importance of EIIB in bacterial virulence regulation. For instance, Arthur et al. [27] demonstrated that the mannose-family *EIIB^MPo^* in *Listeria monocytogenes* exhibits dual regulatory effects on the transcriptional activator ManR, suggesting its potential role in modulating virulence gene expression through mannose metabolism. In *S. agalactiae*, deletion of cel-EIIB in the high-virulence strain THN0901 compromises biofilm formation and reduces invasive and colonization capabilities, likely through modulation of virulence factors such as hemolysin and biofilm-associated genes, ultimately attenuating pathogenicity in *Oreochromis niloticus* [28]. Conversely, cel-EIIB deletion enhances the adhesion of low-virulence strains to tilapia brain cells but significantly impairs biofilm formation in high-virulence strains. Furthermore, multiple virulence-related genes in high- and low-virulence strains exhibit opposing expression patterns, suggesting a differential regulatory role of cel-EIIB depending on strain virulence [29]. Similarly, in *L. monocytogenes*, deletion of EIIB in the highly virulent strain LM928 significantly impaired biofilm formation, intracellular replication, and bacterial loads in murine infection models [30]. We speculate that the EIIB component of the *L. monocytogenes* LIPI-4 (PTS) may exhibit strain-dependent virulence regulation between high- and low-virulence strains.

Therefore, we constructed an *EIIB* gene deletion mutant and its complementation strain derived from the low-virulence strain LM873. These were compared with the existing high-virulence strain LM928 and its corresponding EIIB deletion and complementation strains [30]. Through comprehensive phenotypic analysis, virulence assays, and transcriptional profiling of PTS and virulence-related genes, we aimed to elucidate the role of LIPI-4 (PTS) EIIB in modulating the virulence of *L. monocytogenes*, as well as the mechanistic differences between high- and low-virulence strains. This study provides a new perspective for the research on the pathogenic mechanism of *L. monocytogenes*.

## 2. Materials and Methods

### 2.1. Strains, Plasmids and Cell Lines

All bacterial strains and plasmids used in this study are maintained in our laboratory collection. This includes: the *Listeria monocytogenes* strains LM928 (high-virulence) and LM873 (low-virulence), originally characterized by Xinjiang Academy of Agricultural Reclamation Sciences (Shihezi, China); their derived mutants LM928ΔEIIB and complemented strain CLM928ΔEIIB; the temperature-sensitive shuttle vector pKSV7 and integration plasmid pIMK2; the Escherichia coli DH5α strain was purchased from Transgen Biotech (Beijing, China). All Listeria strains were routinely cultured in brain heart infusion broth (BHI; Hopebio, Qingdao, China) at 37 °C, while *E. coli* was grown in Luria–Bertani medium (Hopebio, Qingdao, China) with appropriate antibiotic selection.

All cell lines were preserved and cultured in our laboratory. Human chorionic carcinoma cell JEG-3 in MEM medium (Procell, Wuhan, China), containing 10% fetal bovine serum (FBS) (ExcellBio, Shanghai, China), cultured under 5% CO_2_. Human colorectal adenocarcinoma cell Caco-2 is cultured in DMEM medium (Gibco, Grand Island, NY, USA), containing 20% fetal bovine serum (FBS), under 5% CO_2_. Mouse mononuclear macrophage leukemia cells, RAW264.7, were cultured in DMEM (10% FBS) media, respectively, under 5% CO_2_.

### 2.2. Animals

ICR mice of 6 to 8 weeks old were purchased from SPF Biotechnology (Beijing, China, https://spfbiotech.com, (accessed on 20 March 2024)). Throughout the experiment, mice were kept under controlled humidity (40–70%) and temperature (21–26 °C). All animal experimental programs comply with the provisions of the National Guidelines for Foster Care and Care of Experimental Animals (China). All animal experiments were approved by the Bioethics Committee of Shihezi University (Approval Number: A2021-26; Approval date: 10 March 2021).

### 2.3. Primers

The primers were designed using the LM873 genome of *L. monocytogenes* strain LM873 in GenBank (accession number NZ_CP046478.1) using Oligo 6.0 software (Supplementary Table S1). The upstream homologous arm of the *EIIB* gene was amplified with primers F1 and R1, and the downstream homologous arm was amplified with primers F2 and R2. The deletion strain was verified using D-F and D-R primers. Use *EIIB* gene amplification primers C-F and C-R to construct complementary strains.

### 2.4. Construction of Mutants and Complement Strains

The deletion mutant LM873ΔEIIB and complemented strain CLM873ΔEIIB were constructed following previously described methodologies [30]. The complement strain CLM873ΔEIIB was constructed using the previously reported strong constitutive promoter P_help_ to enhance gene expression. The recombinant plasmid pIMK2-EIIB was electroporated into competent cells. The LM873ΔEIIB and CLM873ΔEIIB strains were identified by PCR amplification and sequencing. For details of the reaction system and reaction conditions, please refer to Supplementary Tables S2 and S3.

### 2.5. Evaluation of In Vitro Growth in Listeria monocytogenes Strains

The growth kinetics of six *L. monocytogenes* strains (LM873, LM873ΔEIIB, CLM873ΔEIIB, LM928, LM928ΔEIIB, and CLM928ΔEIIB) were evaluated according to a previously established method [31]. Overnight cultures grown in BHI medium at 37 °C with shaking were adjusted to an OD_600_ of 0.5 using fresh BHI. These bacterial suspensions were then diluted 1:100 into new BHI medium and incubated under the same conditions. Bacterial growth was monitored by measuring the OD_600_ at 2 h intervals for a 14 h period using a microplate reader (BioTek, Winooski, VT, USA). Three independent biological replicates were performed for each strain.

### 2.6. Quantitation of Hemolytic Activity by Microplate Technique

Hemolytic activity was determined as described previously [32]. Six strains of LM873, LM873ΔEIIB, CLM873ΔEIIB, LM928, LM928ΔEIIB, and CLM928ΔEIIB were cultured in BHI medium continuously for 12 to 14 h. Use BHI medium to adjust their OD_600_ to 0.6, and then centrifuge at 8000 rpm for 5 min. The hemolytic reaction was initiated by adding 100 μL of 1% sheep erythrocyte suspension (in PBS) to each well, with sterile BHI serving as the negative control. After 2 h incubation at 37 °C, 100 μL of supernatant was carefully aspirated (avoiding erythrocyte disturbance) for spectrophotometric measurement at 550 nm. All assays were performed in triplicate with independent biological replicates.

### 2.7. Assessment of Biofilm Formation Ability

Biofilm formation capacity was quantified using a microtiter plate assay as previously described [25] with modifications. Individual colonies of six strains of LM873, LM873ΔEIIB, CLM873ΔEIIB, LM928, LM928ΔEIIB, and CLM928ΔEIIB were picked and cultured in BHI medium at 37 °C for 12–14 h in a shaking incubator. Subsequently, each well of the 96-well flat-bottomed polystyrene microtiter plates was filled with 150 μL of BHI liquid medium, and 50 μL of bacterial culture medium was added. The OD_600_ value was approximately 0.2, and 8 replicate wells were set at each time. Following static incubation at 37 °C for specified durations (8–72 h), biofilms were processed sequentially: non-adherent cells were removed by PBS washing (3×), methanol-fixed for 30 min, stained with 0.1% (*w*/*v*) crystal violet (100 μL/well, 30 min), and thoroughly rinsed with PBS. For quantification, crystal violet was solubilized in 95% ethanol (30 min), and absorbance was measured at 600 nm using a microplate reader.

### 2.8. RT-qPCR

Six strains of LM873, LM873ΔEIIB, CLM873ΔEIIB, LM928, LM928ΔEIIB, and CLM928ΔEIIB were shaken in BHI medium at 37 °C for 12–14 h. After washing PBS, it was quickly frozen with liquid nitrogen, ground into powder in a mortar. Total RNA was extracted using an RNA extraction kit (TransGen Biotech, Beijing, China), followed by reverse transcription to obtain the corresponding cDNA. Six main genes that make up the PTS were tested, including *Lm4b_02324*, *Lm4b_02325*, *Lm4b_02326*, *Lm4b_02327*, *Lm4b_02328*, *Lm4b_02329.* Virulence and biofilm-related gene transcription were tested, including *hly*, *inlA*, *inlB*, *inlC*, *mpl*, *actA*, *plcA*, *plcB*, *prfA*, *agrA*, *agrB*, *agrC*, *degU*, *flaA*, *mogR*, *motA* and *motB*. To analyze flagellar motility-associated gene expression, single colonies of six bacterial strains were inoculated in BHI and cultured statically at 30 °C for 14 h. Total RNA was extracted using the aforementioned protocol and subsequently reverse transcribed to generate cDNA. We then quantified the transcriptional levels of twelve motility-related genes: *mogR*, *gmaR*, *flaA*, *fliF*, *fliM*, *fliN*, *flhA*, *flhB*, *fliI*, *fliP*, *flgE*, and *flgK*. The housekeeping gene *gyrB* was used as an internal control for normalization. The reaction steps were carried out as described previously [30]. After data collection, relative expression levels were calculated by the threshold cycle (2^−ΔΔCT^) method [33]. Targeted amplification of virulence genes was conducted using primer pairs specified in Supplementary Table S4, with three technical replicates carried out for each assay.

### 2.9. Detection of In Vitro Motor Ability of Bacterial Strains

Evaluation of in vitro motor ability according to the previously described method [34]. Six strains of LM873, LM873ΔEIIB, CLM873ΔEIIB, LM928, LM928ΔEIIB, and CLM928ΔEIIB were shaken and cultured at 37 °C until the OD_600_ were 0.6. The bacterial solutions were dipped in sterilized toothpicks and inoculated on TSA semi-solid plates (containing 2% NaCl, 0.25% Agar, and 1% Tryptone) medium. The diameters of the movement circles of each bacteria were measured after standing culture at 30 °C for 36 h. The experiment was repeated four times.

### 2.10. Determination of Infection Capacity of L. monocytogenes

Established methods [30,35] were adhered to. The JEG-3 and RAW264.7 cell lines were cultured in MEM and DMEM medium supplemented with 10% FBS and 1% penicillin-streptomycin solution (Solarbio, Beijing, China). The Caco-2 cell lines were cultured in DMEM medium supplemented with 1% P/S (penicillin-streptomycin) and 20% fetal calf serum. During bacterial infection assays, extracellular bacteria were eliminated by treatment with gentamicin (100 μg/mL) in the appropriate complete medium. Intracellular bacterial replication was then assessed under continuous antibiotic pressure using a lower concentration of gentamicin (10 μg/mL) in the corresponding complete medium to selectively inhibit extracellular bacterial proliferation. The collected samples were diluted using a serial dilution method [36] and were then used to coat a BHI agar plate. After allowing 24–36 h for colony development at optimal growth conditions, plate counts were performed. Data represent mean values from three independent experimental repetitions.

### 2.11. RT-qPCR Detects the Transcription Level of the Main Virulence Gene After Strain Infecting JEG-3

LM873, LM873ΔEIIB, CLM873ΔEIIB, LM928, LM928ΔEIIB, and CLM928ΔEIIB were cultured in BHI liquid medium to logarithmic growth phase and then infected JEG-3 cells, respectively. After culture at 37 °C for 1 h in 5% CO_2_, complete medium containing a final concentration of 100 µg/mL gentamicin was added, and then placed at 37 °C, and continued to culture at 5% CO_2_ for 1 h, the cells were washed twice with PBS, complete medium containing a final concentration of 10 µg/mL gentamicin was added, and then placed at 37 °C. Cultured in a 5% CO_2_ incubator, washed twice in PBS for another 11 h, and then digested with trypsin to collect the cells, quickly frozen with liquid nitrogen, ground into powder in a mortar, extracted bacterial RNA with an RNA extraction kit (TransGen Biotech, China), and reverse transcribed to obtain corresponding cDNA. The RT-qPCR was used to detect the transcriptional regulation of 11 virulence genes: *actA*, *hly*, *inlA*, *inlB*, *plcA*, *plcB*, *prfA*, *iap*, *inlC*, *inlP* and *mpl*. The reaction procedures and analysis methods are as described above. The primer sequences of the virulence gene of the purpose are shown in Supplementary Table S4. This experiment was repeated three times.

### 2.12. Measurement of Bacterial Burden in Mice

The experimental procedures were adapted from previously established methodology [33], with modifications to the infectious dose parameters. Six strains (LM873, LM873ΔEIIB, CLM873ΔEIIB, LM928ΔEIIB, and CLM928ΔEIIB) were cultured overnight in BHI at 37 °C with shaking. The OD_600_ of the cultures was adjusted to 0.6 (corresponding to ~10^9^ CFU/mL). Bacteria were harvested by centrifugation at 8000 g for 5 min, washed once with sterile phosphate-buffered saline (PBS), and resuspended in PBS for subsequent infections. Infection doses were determined based on established median lethal dose (LD_50_) values: 10^8.6^ CFU for LM873 (low-virulence lineage) versus 10^7.2^ CFU for LM928 (high-virulence lineage) [12]. To ensure cross-strain comparability while maintaining infection efficacy, we employed the following dose regimens: low-virulence group (LM873 and derived mutants): 0.2 mL of 10^7^ CFU suspension (sub-LD50 dose achieving consistent infectivity); High-virulence group (LM928 and derived mutants): 0.2 mL of 10^5^ CFU suspension (dose validated in prior virulence studies [30]). All infections were administered via intraperitoneal injection using sterile techniques. Female ICR mice aged 6–8 weeks (16–19 g body weight) were randomly divided into 7 groups, including PBS, LM873, LM873ΔEIIB, CLM873ΔEIIB, LM928, LM928ΔEIIB, and CLM928ΔEIIB groups, with 6 mice in each group. All animals received 0.2 mL intraperitoneal inoculations of respective bacterial suspensions. Body weights were monitored daily for 72 h post-infection. At the 72 h endpoint, sterile dissections were performed to collect hepatic, splenic, and cerebral tissues. The harvested organs were weighed, homogenized in PBS, serially diluted, and plated on BHI agar plates to quantify the bacterial burden (37 °C, 24–36 h). The results are expressed as colony-forming units per gram of tissue (CFU/g).

### 2.13. Statistical Analysis

Statistical analysis was performed using SPSS 20.0 software (IBM, Armonk, NY, USA), with a significance level set at *p* < 0.05. Data were analyzed using either one-way or two-way analysis of variance (ANOVA) as appropriate to the experimental design. For the biofilm formation assays involving multiple strains and time points, two-way ANOVA was used to evaluate the main effects and interactions, followed by Tukey’s HSD post hoc tests for multiple comparisons. One-way ANOVA and Student’s *t*-test were used for other experiments.

## 3. Results

### 3.1. Construction of LM873EIIB Deletion Strain and Complement Strain

**Figure 1:**
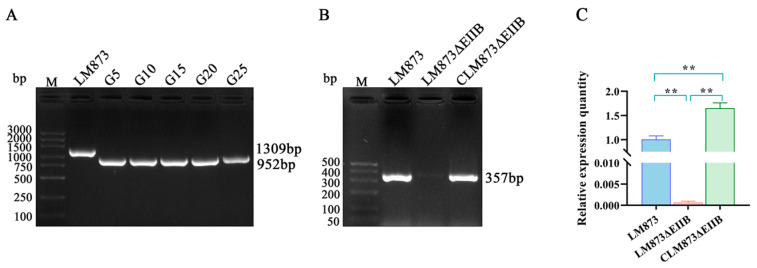
Successful construction of EIIB deletion and complementation strains in LM873. (**A**) The deletion mutants were verified using EIIB-specific primer pair D-F/D-R. Genetic stability of LM873ΔEIIB was confirmed through serial passage analysis, with PCR validation performed at 5-generation intervals (G5, G10, G15, G20, and G25) up to 25 generations. (**B**) PCR verification of the EIIB gene in complementation strain using EIIB-specific primers C-F/C-R. (**C**) Identification of LM873∆EIIB and CLM873∆EIIB strains by RT-qPCR. Values represent the mean ± SEM (*n* = 3), ** *p* < 0.01.

The deletion strain LM873ΔEIIB was cultured at 37 °C for 25 generations, with verification performed every 5 generations using primers D-F/D-R (Figure 1A). Amplification of a 952 bp fragment from the deletion strain confirmed the stable inheritance of the ΔEIIB mutation. Additionally, a 357 bp fragment was amplified from the complemented strain CLM873ΔEIIB using primers C-F/C-R (Figure 1B). Sequencing analysis validated the PCR products from both LM873ΔEIIB and CLM873ΔEIIB. The expression of the *EIIB* gene in the strains LM873, LM873ΔEIIB, and CLM873ΔEIIB was assessed by RT-qPCR. As shown in Figure 1C, EIIB expression was abolished in the LM873ΔEIIB mutant compared to the LM873. In contrast, gene expression was successfully restored in the complemented strain CLM873ΔEIIB, where it reached a significantly higher level. These results demonstrate the successful construction and genetic stability of both mutant strains.

### 3.2. Test Results of in Vitro Growth Ability of Each Strain

**Figure 2:**
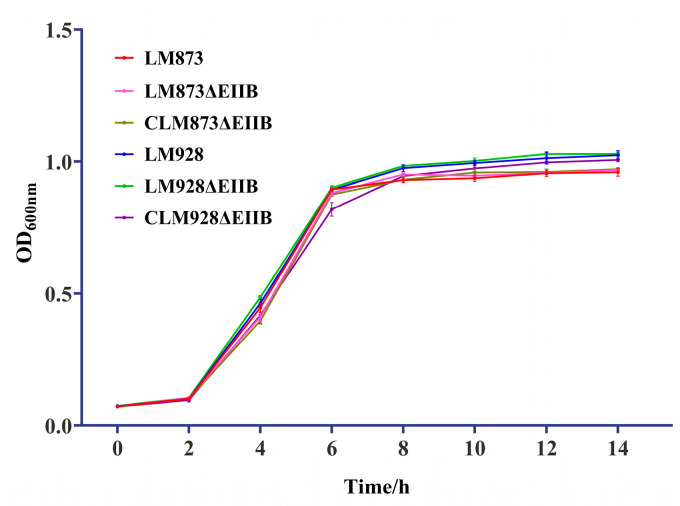
Growth curves of six *L. monocytogenes* strains in BHI medium at 37 °C. Values represent the mean ± SEM (*n* = 3).

Growth curve analysis of six *Listeria monocytogenes* (*L. monocytogenes*) strains in BHI medium revealed three distinct phases: an adaptation phase (0–2 h), followed by logarithmic growth (2–8 h), and finally a stationary phase (8–14 h) (Figure 2). Both the low-virulence strains (LM873, LM873ΔEIIB, CLM873ΔEIIB) and the high-virulence group (LM928, LM928ΔEIIB, CLM928ΔEIIB) exhibited nearly identical growth kinetics, particularly during the exponential phase. Therefore, the EIIB mutation has no significant impact on bacterial growth under the tested conditions.

### 3.3. Test Results of In Vitro Hemolytic Ability of Each Strain

**Figure 3:**
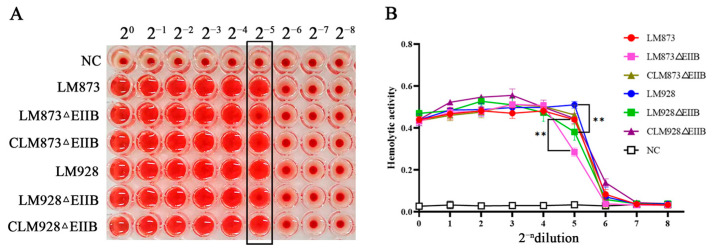
In vitro hemolytic activity of *L. monocytogenes* strains. (**A**) Six strains were individually cultured in BHI medium at 37 °C for 12–14 h. Bacterial suspensions were adjusted to OD_600_ = 0.6 using fresh BHI medium. After centrifugation, 100 μL of each supernatant was transferred to V-bottom wells of a 96-well plate, with uninoculated BHI medium serving as the negative control. An equal volume of 1% sheep red blood cell (sRBC) suspension in PBS was added to each well, followed by incubation at 37 °C for 2 h before imaging. The black box in the figure indicates the 2^−5^ dilution factor, where distinct differences in hemolytic activity between the bacterial strains are observed. (**B**) Quantitative analysis: 100 μL of supernatant was collected for OD_550_ measurement to determine hemolytic activity. Values represent the mean ± SEM (*n* = 3), ** *p* < 0.01.

Hemolytic activity was assessed for six *L. monocytogenes* strains. All strains exhibited hemolytic activity compared to the negative control (NC) (Figure 3A). The hemolytic capacity decreased progressively with increasing dilution. Within the 2^−5^ dilution range, neither LM873ΔEIIB nor LM928ΔEIIB achieved complete erythrocyte lysis. Notably, compared to their wild-type counterparts, both ΔEIIB mutants showed significantly reduced hemolytic activity at this dilution range (*p* < 0.01; Figure 3B). Furthermore, the low-virulence strain LM873 demonstrated weaker hemolytic activity than the high-virulence strain LM928 (Figure 3B).

### 3.4. Experimental Results of Biomembrane Forming Ability

**Figure 4:**
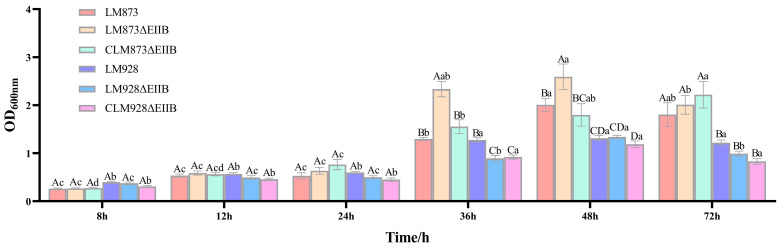
Deletion of EIIB differentially modulates biofilm formation in high- and low-virulence strains. The biofilm formation capacity of the strains was quantified using microtitre plate assays, with optical density at 600 nm serving as the quantitative measurement. Data are from one representative experiment shown as mean ± SEM of *n* = 8 technical replicates. Two-way ANOVA demonstrated significant main effects of strain (F[5,204] = 52.91, *p* < 0.001), time (F[5,204] = 258.5, *p* < 0.001), and strain × time interaction (F[25,204] = 10.90, *p* < 0.001). Distinct uppercase letters (A, B, C, etc.) denote significant inter-strain variations at matched time points (*p* < 0.05), while different lowercase letters (a, b, c, etc.) indicate significant temporal differences within individual strains (*p* < 0.05); bars that share a common letter are not significantly different (*p* > 0.05).

In the biofilm formation assays, the biofilm-forming capabilities exhibited temporal variations among the strains. At 48 and 72 h (Figure 4), the low-virulence strain LM873 formed significantly more biofilm than the high-virulence strain LM928 (*p* < 0.05). Notably, significant inter-strain variations were observed at 36 and 48 h: The low-virulence strain LM873ΔEIIB exhibited significantly enhanced biofilm formation compared to both the wild-type LM873 and complemented strain CLM873ΔEIIB (*p* < 0.05). Conversely, the high-virulence strain LM928ΔEIIB showed a marked reduction in biofilm biomass relative to its wild-type counterpart LM928 at 36 h (*p* < 0.05), while no statistically significant difference was detected when compared with the complemented strain CLM928ΔEIIB (*p* > 0.05). No significant differences were detected at other time points (*p* > 0.05).

### 3.5. Quantitative Analysis of PTS, Virulence, and Biofilm Genes mRNA Levels in L. monocytogenes

**Figure 5:**
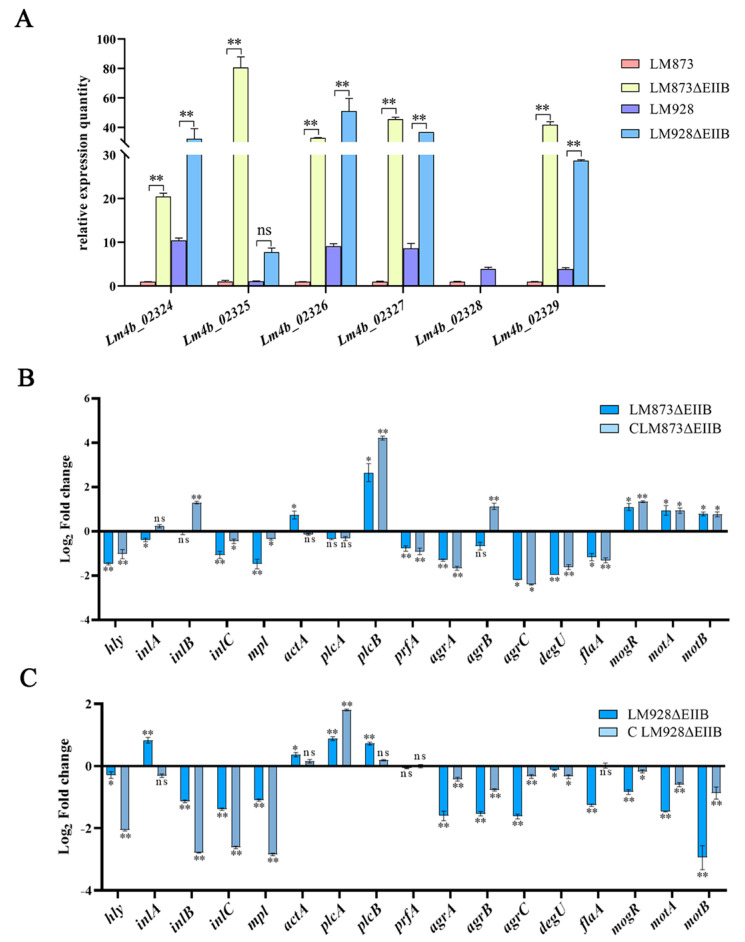
Impact of *EIIB* gene deletion on PTS components, virulence, and biofilm-related gene expression in low- and high-virulence *L. monocytogenes*. (**A**) The expression of PTS components. (**B**) Transcription levels of virulence genes in LM873ΔEIIB and CLM873ΔEIIB compared with LM873. (**C**) Transcription levels of virulence genes in LM928ΔEIIB and CLM928ΔEIIB compared with LM928. The values represent the mean ± SEM (*n* = 3). ns, no significance; * *p* < 0.05, ** *p* < 0.01.

The six major components of the PTS and 17 virulence- and biofilm-related genes in *L. monocytogenes* were quantified by RT-qPCR. As shown in Figure 5A, compared to the respective wild-type strains, deletion of the *EIIB* gene significantly upregulated the expression of *Lm4b_02324*, *Lm4b_02326*, *Lm4b_02327*, and *Lm4b_02329* (*p* < 0.01). Furthermore, the *Lm4b_02325* gene was highly significantly upregulated in the low-virulence strain LM873 (*p* < 0.01) but showed no significant change in the high-virulence strain LM928 (*p* > 0.05).

For the low-virulence strain LM873, EIIB deletion significantly upregulated the transcription of virulence genes *actA*, *plcB*, *mogR*, *motA*, and *motB* (*p* < 0.05), while significantly downregulating *inlA*, *agrC*, and *flaA* (*p* < 0.05). Notably, *hly*, *inlC*, *mpl*, *prfA*, *agrA*, and *degU* showed highly significant downregulation (*p* < 0.01). No significant changes were observed for *inlB*, *plcA*, or *agrB* (*p* > 0.05) (Figure 5B). In the high-virulence strain LM928, EIIB deletion significantly upregulated *actA* (*p* < 0.05) and dramatically upregulated *inlA*, *plcA*, and *plcB* (*p* < 0.01). While *hly* and *degU* were significantly downregulated (*p* < 0.05), *inlB*, *inlC*, *mpl*, *agrA*, *agrB*, *agrC*, *flaA*, *mogR*, *motA*, and *motB* showed highly significant downregulation (*p* < 0.01). *prfA* expression remained unchanged (*p* > 0.05) (Figure 5C). Comparative analysis revealed that EIIB deletion consistently upregulated *actA* and *plcB* in both strains, while downregulating *hly*, *degU*, *inlC*, *mpl*, *agrA*, *agrC*, and *flaA*. Strain-specific effects included: downregulation of *inlB* and *agrB* only in LM928ΔEIIB; upregulation of *plcA* exclusively in LM928ΔEIIB; downregulation of *prfA* exclusively in LM873ΔEIIB; and strikingly opposite expression patterns for *inlA*, *mogR*, *motA*, and *motB* between strains. Quantitatively, EIIB deletion upregulated 29.4% (5/17) of virulence genes in LM873 but downregulated 52.9% (9/17). In LM928, it upregulated 23.5% (4/17) but downregulated 70.5% (12/17) of virulence genes.

### 3.6. In Vitro Motor Ability Detection Results of Each Strain

**Figure 6:**
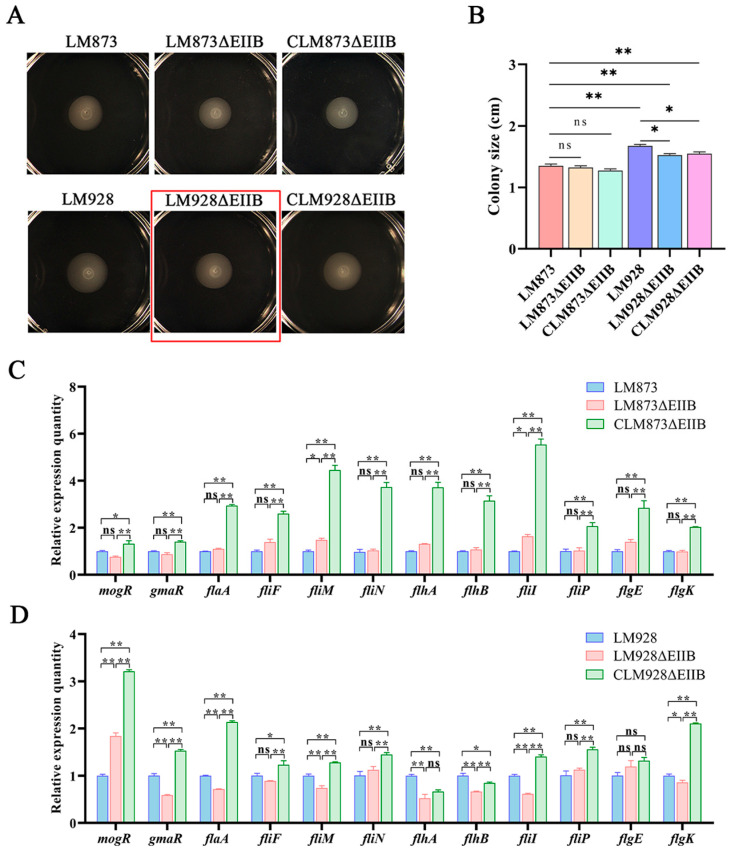
The EIIB deletion specifically impaired motility solely in the high-virulence strain LM928. (**A**) Bacterial migration rings were observed following 36 h static incubation at 30 °C on TSA plates, with red rectangles highlighting significant alterations in the motility zones. (**B**) Quantitative analysis of motility zone diameters (mean ± SEM, *n* = 4). RT-qPCR analysis of flagellar motility-related genes showing: (**C**) mRNA levels in LM873ΔEIIB and the complemented strain CLM873ΔEIIB, normalized to wild-type LM873; (**D**) mRNA levels in LM928ΔEIIB and complemented strain CLM928ΔEIIB, normalized to wild-type LM928. Data are presented as mean ± SEM (*n* = 3). Significance levels: ns, not significant; * *p* < 0.05, ** *p* < 0.01.

Since the most significant differences in biofilm formation between wild-type and EIIB-deficient strains were observed at 36 h in the previous experiment, we assessed bacterial motility in vitro by measuring the migration zone diameters after 36 h of static culture at 30 °C. The results revealed that low-virulence strains exhibited significantly smaller migration zones compared to high-virulence strains (*p* < 0.05) (Figure 6A,B). Notably, while LM873ΔEIIB showed no significant difference in migration zone diameter compared to both LM873 and its complemented strain (*p* > 0.05), LM928ΔEIIB displayed a significantly reduced migration zone compared to LM928 (*p* < 0.05), with no statistically significant difference observed when compared to its complemented strain CLM928ΔEIIB (*p* > 0.05).

The expression levels of 12 flagellar motility-associated genes were analyzed by RT-qPCR. Comparative assessment revealed distinct regulatory patterns between the two mutant strains. In LM873ΔEIIB versus LM873 (Figure 6C), significant upregulation (*p* < 0.05) was observed for *fliM* and *fliI*, whereas the remaining ten genes (*mogR*, *gmaR*, *flaA*, *fliF*, *flgE*, *flgK*, *flhA*, *flhB*, *fliP*, and *fliN*) exhibited no significant differential expression (*p* > 0.05), accounting for 83.3% (10/12) of the tested genes. In contrast, LM928ΔEIIB relative to LM928 (Figure 6D) displayed significant upregulation of *mogR* (*p* < 0.05), while seven genes (*gmaR*, *flaA*, *fliM*, *fliI*, *flhA*, *flhB*, and *flgK*) were markedly downregulated (*p* < 0.01), representing 58.3% (7/12) of the total. The expression levels of the remaining four genes (*fliF*, *fliN*, *fliP*, and *flgE*) remained statistically unaltered (*p* > 0.05).

### 3.7. Strain-Specific Impacts of EIIB Deletion on Host Cell Infection

**Figure 7:**
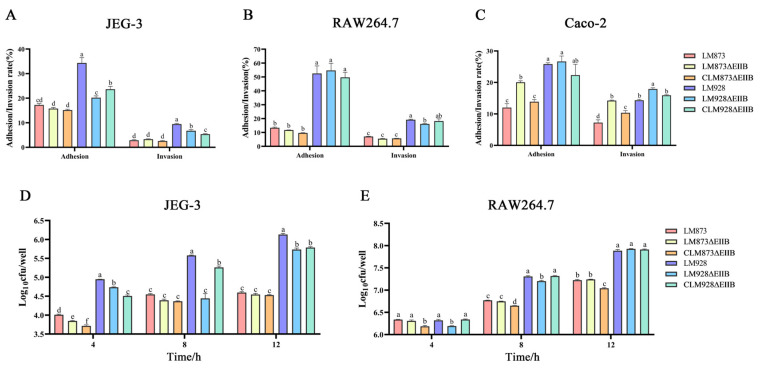
Comparative analysis of bacterial adhesion, invasion, and intracellular proliferation capacities. (**A**–**C**) Adhesion was quantified by enumerating cell-associated bacteria 1 h post-infection, while invasion rates were assessed immediately after an additional 1 h treatment with 100 μg/mL gentamicin (total 2 h post-infection). (**D**,**E**) Intracellular proliferation was measured from the 0 h time point (defined as initiation of 100 μg/mL gentamicin treatment), with infected cells subsequently maintained in medium containing 10 μg/mL gentamicin; samples were collected at 4, 8, and 12 h time points. The values represent the mean ± SEM (*n* = 3). Distinct letters denote significant inter-strain variations (*p* < 0.05); bars that share a common letter are not significantly different (*p* > 0.05).

JEG-3, RAW264.7 and Caco-2 cells were infected with bacteria at multiplicity of infection values of 1, 1 and 10, respectively. The results demonstrated that the high-virulence strains exhibited significantly higher infectivity than the low-virulence strains (*p* < 0.05). For JEG-3 cells (Figure 7A,D), LM873ΔEIIB showed no significant differences in adhesion or invasion rates compared to its wild-type and complemented strains (*p* > 0.05). However, the intracellular bacterial load of LM873ΔEIIB was significantly lower than that of LM873 only at 4 h (*p* < 0.05), with no significant differences observed at 8 and 12 h (*p* > 0.05). In contrast, LM928ΔEIIB exhibited significantly reduced adhesion and invasion rates compared to LM928 (*p* < 0.05), and its intracellular bacterial load was significantly lower than that of LM928 at 4, 8, and 12 h (*p* < 0.05). For RAW264.7 cells (Figure 7B,E), LM873ΔEIIB displayed no significant differences in adhesion rate, invasion rate, or intracellular bacterial load compared to its wild-type and complemented strains (*p* > 0.05). LM928ΔEIIB showed a significantly decreased invasion rate compared to LM928 (*p* < 0.05), while its adhesion rate remained unchanged (*p* > 0.05). Additionally, the intracellular bacterial load of LM928ΔEIIB was significantly lower than that of LM928 at 4 and 8 h (*p* < 0.05), but no significant difference was observed at 12 h (*p* > 0.05). For Caco-2 cells (Figure 7C), LM873ΔEIIB exhibited significantly higher adhesion and invasion rates compared to its wild-type and complemented strains (*p* < 0.05). In contrast, LM928ΔEIIB showed no significant difference in adhesion rate (*p* > 0.05) but a significantly increased invasion rate relative to its wild-type and complemented strains (*p* < 0.05).

### 3.8. Quantitative Analysis of Virulence Gene Expression After JEG-3 Infection

**Figure 8:**
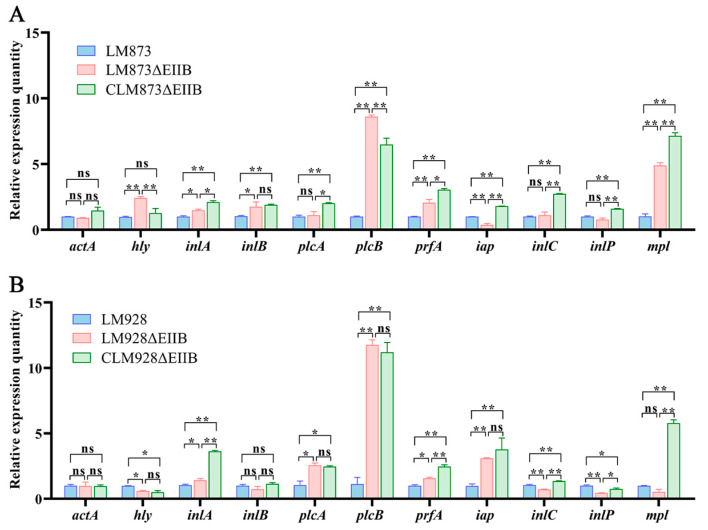
RT-qPCR assays. The cDNA templates of six strains of bacteria collected after infecting JEG-3 cells were subjected to RT-qPCR to determine fold changes in virulence genes. (**A**) Expression of virulence gene transcription levels of LM873ΔEIIB and CLM873ΔEIIB compared with LM873. (**B**) Expression of virulence gene transcription levels of LM928ΔEIIB and CLM928ΔEIIB compared with LM928. The values represent the mean ± SEM (*n* = 3). ns, no significance; * *p* < 0.05, ** *p* < 0.01.

Total bacterial RNA was extracted from JEG-3 cells infected for 12 h with LM873, LM873ΔEIIB, CLM873ΔEIIB, LM928, LM928ΔEIIB, or CLM928ΔEIIB, followed by RT-qPCR analysis of 11 major virulence genes. In the low-virulence strain LM873 (Figure 8A), EIIB deletion significantly upregulated the transcription of *inlA* and *inlB* (*p* < 0.05), markedly increased expression of *hly*, *plcB*, *prfA*, and *mpl* (*p* < 0.01), while significantly downregulating *iap* (*p* < 0.01), with no significant changes observed in *actA*, *plcA*, *inlC*, or *inlP* expression (*p* > 0.05). For the high-virulence strain LM928 (Figure 8B), EIIB deletion led to significant upregulation of *inlA*, *plcA* and *prfA* (*p* < 0.05); dramatic increase in *plcB*, *iap* expression (*p* < 0.01); along with significant downregulation of *hly* (*p* < 0.05) and marked reduction in *inlC* and *inlP* transcription (*p* < 0.01); while *actA*, *inlB*, and *mpl* remained unchanged (*p* > 0.05).

Comparative analysis revealed that the expression levels of eight genes (*hly*, *inlB*, *plcA*, *iap*, *inlC*, *inlP*, and *mpl*) were inconsistent between the LM873ΔEIIB and LM928ΔEIIB mutant strains compared to their respective wild-type counterparts. Quantitatively, EIIB deletion upregulated 54.5% (6/11) and downregulated 9.1% (1/11) of virulence genes in LM873, while in LM928 it upregulated 36.4% (4/11) and downregulated 36.4% (4/11) of virulence genes.

### 3.9. Determination Results of Bacterial Load in Mouse Organs

**Figure 9:**
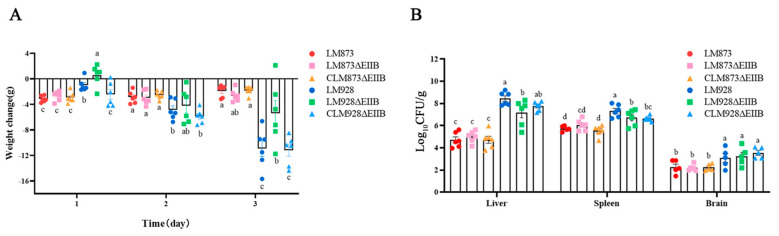
The deletion of EIIB specifically reduced colonization of the high-virulent strain LM928 in mouse liver and spleen. Mice were intraperitoneally injected with 10^7^ CFU of LM873, LM873ΔEIIB, or CLM873ΔEIIB, or with 10^5^ CFU of LM928, LM928ΔEIIB, or CLM928ΔEIIB. (**A**) Body weights were measured daily (days 0–3), with values representing weight changes relative to day 0. (**B**) On day 3, mice were euthanized, and liver, spleen, and brain tissues were collected. Tissues were weighed, homogenized, serially diluted, and plated on BHI agar for CFU enumeration after 24–36 h incubation at 37 °C. Data are presented as Log_10_ CFU per gram of tissue. Values represent mean ± SEM (*n* = 6). Different letters above bars indicate statistically significant differences between groups (*p* < 0.05).

Mice were intraperitoneally injected with bacterial suspensions of LM873, LM873ΔEIIB, CLM873ΔEIIB, LM928, LM928ΔEIIB or CLM928ΔEIIB. Following infection, experimental mice exhibited clustering behavior, lethargy, trembling, and partially closed eyes. Daily weight measurements revealed no significant differences in body weight changes over three days between LM873ΔEIIB and either LM873 or CLM873ΔEIIB groups (*p* > 0.05) (Figure 9A). In contrast to mice infected with either LM928 or CLM928ΔEIIB strains, those challenged with LM928ΔEIIB showed less weight loss on day 1 post-infection. However, by day 3, these animals developed significantly reduced body weight (*p* < 0.05) compared to both control groups.

Organ bacterial loads were quantified by plating homogenates of liver, spleen, and brain tissues collected on day 3 (Figure 9B). LM873ΔEIIB demonstrated comparable bacterial colonization in liver, spleen, and brain to both LM873 and CLM873ΔEIIB (*p* > 0.05). In contrast, the high-virulence strain LM928ΔEIIB displayed significantly attenuated bacterial loads relative to its parental LM928 strain, exhibiting a 1.25 log_10_ reduction in hepatic colonization (from 8.44 ± 0.60 to 7.19 ± 1.17 log_10_ CFU/g; *p* < 0.05) and a 0.6 log_10_ decrease in splenic burden (from 7.32 ± 0.56 to 6.72 ± 0.71 log_10_ CFU/g; *p* < 0.05). No statistically significant difference was detected in cerebral colonization between LM928ΔEIIB and LM928 (*p* > 0.05).

## 4. Discussion

The bacterial phosphotransferase system (PTS) is a complex, multicomponent signal transduction system critical for carbohydrate uptake and phosphorylation, while also regulating carbon and nitrogen metabolism in response to sugar availability [37]. In *Listeria monocytogenes*, LIPI-4 is a strain-specific virulence factor belonging to the cellobiose family of the phosphoenolpyruvate (PEP)-dependent PTS. Within this system, the membrane-associated component EIIB, which facilitates sugar phosphorylation, acts as a critical subunit of the sugar-specific EII complex [11,19].

Growth experiments performed by Xie et al. [25] revealed no significant difference between the wild-type strain and the Δcel-EII mutant of *Streptococcus agalactiae* in BHI medium. Similarly, Zeng and Burne [38] demonstrated that mutations in individual components of the sucrose-starch metabolism PTS (scr-PTS) EII complex in *Streptococcus mutans* did not cause noticeable growth defects. Previous studies also indicated that deletion of EIIB did not significantly affect the growth rate of the highly virulent L. monocytogenes strain LM928 [30]. Consistent with these findings, our results indicated no notable growth differences between the EIIB deletion mutant and the wild-type or complemented control strains, irrespective of virulence level (Figure 2), further supporting that EIIB is nonessential for in vitro proliferation of *L. monocytogenes*.

Xie et al. [25] proposed that the role of the cel-EII complex in *S. agalactiae* may stem from complex genetic interactions, with *cel-EIIA*, *cel-EIIB*, or *cel-EIIC* genes potentially contributing to virulence in distinct ways. To explore this further, we analyzed the expression of other major PTS components after EIIB deletion. Our results (Figure 5A) indicated that EIIB deletion significantly upregulated the expression of *Lm4b_02324*, *Lm4b_02326*, *Lm4b_02327*, and *Lm4b_02329*—which encode maltose-6′-beta-glucosidase, uncharacterized PTS-related proteins, EIIA, and EIIC, respectively—compared to their respective wild-type strains. Notably, a key difference between high- and low-virulence strains was observed in the expression of *Lm4b_02325* (encoding a transcriptional antiterminator), which was significantly increased only in the low-virulence strain LM873ΔEIIB, with no change in the high-virulence strain LM928ΔEIIB.

Transcriptional antitermination mechanisms are known to regulate gene expression in *Bacillus subtilis* [39]. Our results are consistent with this mechanism, as deletion of EIIB in the low-virulence LM873 strain led to a marked enhancement in biofilm formation capacity while causing only marginal alterations in cellular infectivity and bacterial load in mouse models—a striking contrast to the significant reductions observed in the high-virulence LM928 strain (Figure 4, Figure 7 and Figure 9). We hypothesize that the low-virulence strain may compensate for virulence defect and restore metabolic fitness through upregulation of antitermination mechanisms, though the precise underlying regulatory pathways require further investigation.

Cao et al. [40] reported that the EIIA protein encoded by cel-PTS positively regulates the virulence of *L. monocytogenes*. However, we found that EIIB deletion upregulated the endogenous *EIIA* gene in both high- and low-virulence strains, suggesting that differences in virulence are unlikely to be due to *EIIA* upregulation. The regulatory interplay among PTS components appears complex and merits further investigation.

Biofilm formation provides *L. monocytogenes* with a survival advantage in food processing environments, representing a primary source of foodborne contamination [41,42]. The PTS has been implicated in biofilm regulation across various pathogens [43,44]. Although prior work showed that EIIB deletion greatly reduced biofilm formation in the high-virulence strain LM928 [30], we found that EIIB deletion enhanced biofilm formation in the low-virulence LM873 while suppressing it in LM928 (Figure 4), highlighting the functional specificity of EIIB across genetic backgrounds. Biofilm formation is a complex biological process regulated by multiple genes involved in signal transduction, extracellular matrix synthesis, quorum sensing (QS), metabolic regulation, and motility [45,46,47,48,49]. Comparative analysis of biofilm-associated gene expression (Figure 5B,C) showed that EIIB deletion downregulated *degU* (a two-component regulatory system gene), *agrA*/*C* (QS genes), and *flaA* (a flagellar structural gene) in both high- and low-virulence strains, while *agrB* was significantly downregulated only in LM928ΔEIIB. These results support the known positive role of the Agr QS system in biofilm formation [50] and correlate *agrB* downregulation with impaired biofilm formation in LM928ΔEIIB.

The strain-specific regulatory role of EIIB was also evident in motility: EIIB deficiency impaired motility only in the high-virulence LM928 at 30 °C (Figure 6A,B), accompanied by *mogR* upregulation and concurrent downregulation of *gmaR* and flagellar structural genes (Figure 6C,D). This supports the established model of MogR-GmaR-mediated regulation of flagellar genes at low temperatures [51], indicating that EIIB may specifically affect motility in high-virulence strains by disrupting the *mogR*/*gmaR* equilibrium. In contrast, the motility-associated genes in low-virulence strain LM873 remained largely unaffected.

It is noteworthy that in the complementary strain CLM873ΔEIIB, the expression of several genes did not fully revert to wild-type levels but instead showed a moderate increase (Figure 6). This observation may be attributed to the use of the strong constitutive promoter P_help_ in the complementation vector, which can drive higher transcriptional activity than the native promoter. Although this led to quantitative differences in mRNA abundance, the key finding is that genetic complementation successfully restored critical phenotypes—including hemolytic activity, biofilm formation, and motility—to levels comparable to the wild-type strain (Figure 3, Figure 4 and Figure 6). This clear functional rescue demonstrates that the regulatory role of EIIB was reconstituted, underscoring its essential role in these pathways. We acknowledge that the use of a strong promoter is a limitation of the current complementation strategy. Future studies involving chromosomal integration of the gene under the control of its native promoter will help to precisely recapitulate the wild-type expression state.

Bacterial adhesion to host epidermal cells is a critical step in colonization and pathogenicity, serving as a prerequisite for subsequent invasion, immune evasion, and proliferation [52]. *L. monocytogenes* can invade placental goblet cells and epithelial folds via transcellular transport, resulting in systemic infection [53]. Accumulating evidence indicates that the PTS plays a pivotal role in modulating bacterial virulence [54,55,56]. Our data show that EIIB deletion significantly impaired the ability of high-virulence LM928 to adhere to and invade human choriocarcinoma (JEG-3) cells (Figure 7A), indicating that EIIB is essential for placental infection by this strain. In RAW264.7 cells, LM928ΔEIIB showed reduced invasion but unchanged adhesion (Figure 7B), implying that the mutation specifically disrupts internalization without affecting initial attachment in phagocytic cells. Intracellular proliferation assays (Figure 7D,E) revealed that EIIB deletion reduced early intracellular replication of low-virulence LM873 in JEG-3 cells, while more profoundly attenuating proliferation of high-virulence LM928 in both JEG-3 and RAW264.7 macrophage cells. Given that intracellular proliferation is closely linked to lipid metabolism—where lipids function as membrane components, energy sources, and signaling molecules [57,58,59]—we propose that EIIB may govern fatty acid synthesis or lipoprotein uptake, with its deletion potentially disrupting membrane biogenesis required for cytoplasmic replication. Interestingly, EIIB loss in high-virulence strains led to opposing infection phenotypes: invasion increased in Caco-2 cells but decreased in JEG-3 cells (Figure 7A,C). This cell type-dependent effect may reflect differences in metabolic environments. Caco-2 cells mimic a carbohydrate-rich intestinal niche [60] that may favor EIIB-deficient mutants due to altered carbon metabolism. In contrast, JEG-3 cells represent a metabolite-dependent placental niche [61] that may be less permissive to infection upon EIIB deletion, possibly due to impaired nutrient utilization. These findings underscore how metabolic adaptations in pathogens modulate host–pathogen interactions based on the infected cell type’s nutritional microenvironment.

Furthermore, EIIB deletion significantly reduced hemolytic activity in both strains (Figure 3A,B). Since bacterial hemolysis aids phagosome escape and intracellular survival [62,63], this reduction is likely relevant to virulence. Hemolytic activity primarily depends on listeriolysin O (LLO), a pore-forming cytolysin encoded by hly [6], facilitating bacterial escape. The master virulence regulator *PrfA* controls *hly* expression [64]. Quantitative PCR analysis revealed that EIIB deletion significantly reduced *hly* gene expression in both high- and low-virulence strains (Figure 5B,C). These findings indicate that reduced *hly* expression directly decreases hemolysin production, implying that EIIB positively regulates *L. monocytogenes* hemolytic capacity. We further observed a strain-specific regulatory phenomenon: the coupling between the expression of PrfA and its core target gene, *hly*, differed between the high- and low-virulence strains. In the low-virulence strain LM873, the deletion of EIIB consistently reduced the transcript levels of both *prfA* and *hly* (Figure 8A), which aligns with the canonical regulatory model. In contrast, within the high-virulence strain LM928, while EIIB deletion significantly diminished *hly* expression, it did not correspondingly reduce the transcriptional level of *prfA* (Figure 8B). Given that hemolytic activity was significantly impaired in both mutants, this transcriptional uncoupling in strain LM928 strongly suggests that the activity of the PrfA protein itself may be subject to post-transcriptional regulation. This idea is supported by established literature indicating that PrfA activity can be modulated post-translationally, for instance, through modifications or cofactor binding [65,66]. As the present study did not provide direct evidence, via Western blotting or activity assays, to confirm alterations in PrfA activity status, this remains a compelling hypothesis for future investigation. Most importantly, this discrepancy underscores a fundamental difference in the architecture of the virulence regulatory network between high- and low-virulence strains: although EIIB deletion ultimately attenuated hemolytic activity in both, the underlying molecular pathways involved are strain-specific.

Notably, deletion of EIIB significantly reduced liver and spleen colonization by the highly virulent strain LM928 compared to its wild-type counterpart, whereas its impact on the low-virulence strain LM873 was not statistically significant (Figure 9B). Previous studies have demonstrated that defects in PTS components significantly impair bacterial virulence and organ colonization in mice [30,67]. RT-qPCR analysis of infected JEG-3 cells (Figure 8B) showed that EIIB deletion markedly attenuated virulence gene expression (*hly*, *inlC*, and *inlP*) only in LM928, correlating with reduced colonization in vivo. These findings suggest divergent virulence regulation between strains, with high-virulence LM928 exhibiting stronger dependence on EIIB for maintaining virulence gene expression and pathogenicity in vivo. We also observed a contextual shift in the expression of the key virulence regulator *prfA*: although EIIB deletion downregulated *prfA* in LM873 under in vitro conditions (Figure 5B,C), both strains showed substantial *prfA* upregulation during JEG-3 infection (Figure 8A,B). Extracellular conditions (e.g., low temperature, abundant oligopeptides and fatty acids) suppress PrfA activity, while the intracellular milieu (e.g., reductive state, nutrient stress, and stress signals) activates it [66]. Henderson et al. [68] identified novel PrfA-regulated genes with differential expression in vitro versus in vivo, supporting the role of complex intracellular cues—including diverse stress signals—in modulating PrfA activation. Our results are consistent with PrfA’s responsiveness to multiple intracellular stimuli, underscoring the ability of *L. monocytogenes* to sense and adapt to host microenvironments to coordinate virulence expression.

The current study builds upon previous work by investigating the role of EIIB, a key component of PTS, in *Listeria monocytogenes* strains with divergent virulence (low-virulence LM873 and high-virulence LM928). We demonstrate that although EIIB deletion does not impair growth, it leads to strain-specific modulation of key virulence phenotypes—including biofilm formation (enhanced in LM873ΔEIIB but attenuated in LM928ΔEIIB), motility, host cell invasion, intracellular replication, and organ colonization in mice—accompanied by significant transcriptional changes in core virulence genes (*prfA*, *hly*). Our work reveals that EIIB exerts complex, strain-dependent regulatory effects on virulence pathways in *L. monocytogenes*, with distinct metabolism–virulence coupling patterns between high- and low-virulence strains. EIIB appears to exert strong positive regulation in the high-virulence LM928, whereas the mechanisms behind certain EIIB-mediated phenotypes in LM873—particularly enhanced biofilm formation—remain unclear.

A key limitation of this study is the lack of direct metabolomic validation. Given that the PTS is a central regulator of carbohydrate sensing and carbon metabolic homeostasis [21], and based on our hypothesis that EIIB deletion alters carbon flux and thereby remodels virulence phenotypes and transcriptional outputs, future work should include systematic metabolomic profiling. Subsequent studies should also examine: the effect of EIIB deletion on lipid metabolism and intracellular proliferation; the role of EIIB in placental tropism using pregnant animal models; and whether functional differences in EIIB between strains reflect distinct selective pressures during ecological adaptation.

## Data Availability

The original contributions presented in the study are included in the article/Supplementary Materials. Further inquiries can be directed to the corresponding authors.

## Supplementary Materials

The following supporting information can be downloaded at: https://www.mdpi.com/article/10.3390/microorganisms13102274/s1. Table S1: The primer sets utilized for strain construction and verification; Tables S2: Splicing with overlap extension PCR reaction system; Tables S3: SOE-PCR reaction conditions; Table S4: The information of RT-qPCR primer specifications.
