# Supplementary material for: The PTS EIIB Component Drives Strain-Specific Virulence in Listeria monocytogenes: Divergent Regulation of Biofilm Formation and Host Infection in High- and Low-Virulence Strains
Source: Microorganisms. 2025 Sep 28;13(10):2274. doi: 10.3390/microorganisms13102274 (PMC12566407; doi:10.3390/microorganisms13102274)
Supplement: Supplementary file 1 [file microorganisms-13-02274-s001.zip › Supplementary Materials.pdf]

Table S1. The primer sets utilized for strain construction and verification. All primers were synthesized by Sangon Biotech, China.

Primers		Sequence（5′→3′）	Restriction site	Amplicon size(bp)
Mutant construction				
Upstream homologous recombination arm primers	F1	*AA*CTGCAGGCCAGTAATGTTTGGTGTTCC	*Pst* I	344
	R1	CTGTAATTCAGTGCCTTCCAGTCTATCGTCCT		
Downstream homologous recombination arm primers	F2	AGGACGATAGACTGGAAGGCACTGAATTACAG		375
	R2	*CG*GAATTCTGATTGCTCTTGTGAACCC	*Eco R* I	
Detecting primers	D-F	GTTACAGGAGCACTCGTTGTC		952(1309)
	D-R	GAGATGTAAGCCTATTCCAAG		
Complementary construction	C-F	*AA*CTGCAGGTAGGAAAGGACGATAGACATGAAA	*Pst* I	357
	C-R	*CCG*CTCGAGTTAGTTTCCCTCCGTCTTAAATGTA	*Xho* I	

Note: The protective bases are set to italic; The restriction enzyme sites are underlined.

Table S2. Splicing with overlap extension PCR reaction system.

Primers	Sequence（5′ →3′ ）	Restriction site	Amplicon size(bp)
One	PCR product purified by the upstream homology	2.0	30.0
	PCR product purified by the downstream homology	2.0	
	Taq Plus Master Mix II	20.0	
	Ultrapure water	6.0	
Two	F1 primers	2.0	35.0
	R2 primers	2.0	
	dNTP (TransGen Biotech, China)	1.0	
	First step product	30.0	

Table S3. SOE-PCR reaction conditions.

Reaction steps	Reaction conditions (℃)	Reaction time (min)	Number of cycles
	95	5.0	1
	95	0.5	
One	58	0.5	18
	72	1.0	
	72	10.0	1
	95	5.0	1
	95	0.5	
Two	58	0.5	35
	72	1.0	
	70	10	1

Table S4. The information of RT-qPCR primer specifications.

Name	Forward primer sequence （5′→3′）	Reverse primer sequence （5′→3′）	Amplicon size(bp)
Lm4b_02324	ATTACTATTGCTGGCGGAGGA	CATACAAACGGATTTCACTTAC	100
Lm4b_02325	AGCCACAGTAGAAGCAAATA	TCATACGCATAATCCGTTA	93
Lm4b_02326	TATTACGCCTGAGTATCCCTAT	CCAAATGACTCGGTTTCTT	104
Lm4b_02327	GAGATGCCTCTTCCTCGTTT	GTTCAATACATGCTTCTGCTT	81
Lm4b_02328	TCTCAATGGCTTATGAACAG	GCAGCGATTAGAATTGGTAC	121
Lm4b_02329	TTTTCTCCACTGACATCCCTA	CGCTACACTGAGCCAAACT	129
actA	CGACATAATATTTGCAGCGAC	CGTGAACTTACTTCACGTGCA	138
agrA	GCAGCCGGACATGAATGG	AACCACGCGGATCAAACTTC	62
agrB	TCGCTCCGGCAGACACA	TTTTTAGTGTTTTCCGGTGTTCTTC	63
agrC	TATTTTGCTAGATAATGCGGTTGAA	CGCGATTCGAATAACTGGATTT	64
degU	ACGCATAGAGAGTGCGAGGTATT	CCCAATTCCGCGGTTACTT	63
flaA	GACTTGTTACAAACAGAGGATTCA	ATTGACGCATACGTTGCAAGAT	67
fliF	TCGAAGGGACACTTTCCAGC	TTGAGGCGGTTCCTGATTCC	195
fliM	TGAGCGAGCGCAGACTTTTA	CAACACTGACAAGCGCCATC	192
fliN	TCGCACGAGAAAAAGCGAAA	CGGAAAAGTGCTTCATTTTGGTC	180
fliI	AAAACGGCTCGATCACTGGT	CTAACGGAGCCGAGGACATC	166
flgE	GCGGTGGCTACTTTCTCCAA	ATTCGCGGGACAAGTCTACG	169
flgK	ACAAGCTGTGGATCAGACGG	AACGAAGCGGATTGTTTGGC	156
flhA	ATGAACTCCTGATGCGCCAA	GTTGTCGTAGCACCCCTTGA	129
flhB	CTTGCGGATGTCGAAGCAAG	TGTCGGGTTGTTCACCACA	157
fliP	TGAATGTGCATGCCGAGAGT	ACAAACAGCGCCACACTAGA	82
gyrB	AGACGCTATTGATGCCGATGA	GTATTGCGCGTTGTCTTCGA	91
gmaR	AGCAAGTTCCATCAACCAAAAG	GTTGAGTTGTCATCGAAAGTAAGC	103
hly	ATTGCGCAACAAACTGAAGC	TCGATTGGCGTCTTAGGACT	110
inlA	AAAGATATAGGCACATTGGCGAG	GACCCGACAGTGGTGCTAGATTA	91
inlB	GTGAAAGAAAAGCACAACCCAAG	TCGCCCCGTTTCCAATAATTAT	94
inlC	AAAACCAAGCATCAACAATACT	TGTTTTTAGATAACAACGAACTC	113
iap	TACTTCTGGCGCACAATA	ATTGTCTTGCGCGTTAAT	148
inlP	CCAACTTCCGACGAC	CTCCAGTGGCATAATTGT	116
mpl	GGCGTTACGCATTATACGC	TATTATCCGGTGTGACGTGG	88
mogR	AACTGCCGAAGAAATCTACCATTT	CGATTCCACCGTGTTCTTCA	68
motA	CAACGCTCGGTGTACTTGGA	TTTCGCCCATCGCATGA	54

motB	TGCAAAAAAATTCGAACAAATGG	CTGCCGCGCCTTCCT	62
plcA	CAACTAGAAGCAGGAATACGGTAC	TGAGTAATCGTTTCTAATACACCTG	116
plcB	TATCAAGCAACAGAAGACATGGT	TGACTATTTTCGGGTAGTCCG	109
prfA	GCCAACCGATGTTTCTGTATCA	TGGTATCACAAAGCTCACGAGT	115
